# Modeling Test and Treatment Strategies for Presymptomatic Alzheimer Disease

**DOI:** 10.1371/journal.pone.0114339

**Published:** 2014-12-04

**Authors:** James F. Burke, Kenneth M. Langa, Rodney A. Hayward, Roger L. Albin

**Affiliations:** 1 Dept. of Neurology, University of Michigan, Ann Arbor, Michigan, United States of America; 2 Robert Wood Johnson Clinical Scholars Program, University of Michigan, Ann Arbor, Michigan, United States of America; 3 Dept. of Internal Medicine, University of Michigan, Ann Arbor, Michigan, United States of America; 4 Center for Clinical Management Research, VAAAHS, Ann Arbor, Michigan, United States of America; 5 Institute for Social Research, University of Michigan, Ann Arbor, Michigan, United States of America; 6 Geriatric Research, Education, and Clinical Center, and Neurology Service, VAAAHS, Ann Arbor, Michigan, United States of America; 7 Michigan Alzheimer Disease Center, University of Michigan, Ann Arbor, Michigan, United States of America; Cuban Neuroscience Center, Cuba

## Abstract

**Objectives:**

In this study, we developed a model of presymptomatic treatment of Alzheimer disease (AD) after a screening diagnostic evaluation and explored the circumstances required for an AD prevention treatment to produce aggregate net population benefit.

**Methods:**

Monte Carlo simulation methods were used to estimate outcomes in a simulated population derived from data on AD incidence and mortality. A wide variety of treatment parameters were explored. Net population benefit was estimated in aggregated QALYs. Sensitivity analyses were performed by individually varying the primary parameters.

**Findings:**

In the base-case scenario, treatment effects were uniformly positive, and net benefits increased with increasing age at screening. A highly efficacious treatment (i.e. relative risk 0.6) modeled in the base-case is estimated to save 20 QALYs per 1000 patients screened and 221 QALYs per 1000 patients treated.

**Conclusions:**

Highly efficacious presymptomatic screen and treat strategies for AD are likely to produce substantial aggregate population benefits that are likely greater than the benefits of aspirin in primary prevention of moderate risk cardiovascular disease (28 QALYS per 1000 patients treated), even in the context of an imperfect treatment delivery environment.

## Introduction

Alzheimer disease (AD) is a largely untreatable major public health problem whose aggregate social costs approximate those of cancer and cardiovascular disease. [1], [2] With AD prevalence rising in both developed and developing nations due to population aging, AD constitutes an urgent global problem. [3], [4] Strong genetic evidence supports the amyloid hypothesis that excessive production or impaired catabolism of amyloidogenic fragments (Aß 40 and A42 peptides) of the amyloid precursor protein (APP) initiate pathogenic cascades causing neuronal dysfunction and degeneration. [5], [6] Trials of anti-amyloid therapies in those with AD, however, have been disappointing,[7], [8] with little evidence of clinical benefit despite some biomarker indications of diminished brain amyloid burden. [9], [10] These disappointing trial outcomes lead to a hypothesis that treatment in symptomatic AD subjects is too late. At the time of diagnosis, considerable neurodegeneration has occurred and Aß peptide has initiated secondary pathogenic cascades unaffected by primary anti-amyloid therapies [2], [11]–[13].

Treating patients prior to the development of overt AD-related symptoms (likely based on biomarker based screening) is a clinical paradigm with at least one close analogue – primary prevention of cardiovascular disease. While primary prevention of cardiovascular disease is thought to be at least partly responsible for major societal declines in cardiovascular mortality,[14] the gains for individual patients are often modest. [4], [15], [16] Over 100 moderate risk patients, for example, need to be treated with aspirin to prevent a single cardiovascular event. [1], [6] Translating a similar primary prevention approach to AD will be more challenging. AD symptoms develop later in life than cardiovascular-related disability, with competing causes of mortality a greater concern. Some treated patients will die before developing AD and would be exposed to risks of therapy only, without receiving benefits of treatment. This challenge is exacerbated by the potentially prolonged interval between treatment initiation and symptom development. For primary prevention of cardiovascular disease, blood pressure treatment reduces individual-level risk by several absolute percentage points [3], [9], [10] within a decade and measurably reduces mortality over intervals as short as two years. [2], [5], [11]–[13] For primary prevention of AD, however, treatment may need to be started as much as 15 years before symptom development – exposing patients to a prolonged window of risk before realizing benefits. Presymptomatic AD screen and treat strategies will have to overcome other distinctive challenges (e.g., lower population prevalence of AD and the need to develop biomarker-based screening tools) as well as challenges in common with cardiovascular disease prevention (e.g., risks of medications in an aging population, medication compliance). These challenges raise the possibility that that even wide implementation of an efficacious presymptomatic AD treatment may fail to deliver the anticipated major societal benefit.

Modeling studies facilitate evaluation of questions not readily measured, but can be approached using reasonable assumptions. Such studies may provide guidance for developing clinical trials and biomarker studies by informing questions such as when presymptomatic treatments should be initiated, what screening strategies are optimal, and what harm magnitudes are acceptable. Modeling is also useful often for initial cost-effectiveness and cost-benefit analyses. In this manuscript, we describe the development of a formal model of presymptomatic AD screening and treatment using Monte Carlo simulation methods to provide a framework for addressing some of these questions. We then apply this model to broadly explore the effect of varying presymptomatic treatment parameters on outcomes. Our findings suggest interesting features of presymptomatic AD screen and treat strategies that may inform trial and biomarker study designs.

## Methods

Our primary goal was to estimate the thresholds of efficacy and harm needed for a novel treatment to deliver aggregate net patient benefit (quality-adjusted life years [QALYs] saved or lost per 1,000 patients screened) in a population of patients at risk for developing AD.

**Figure 1:**
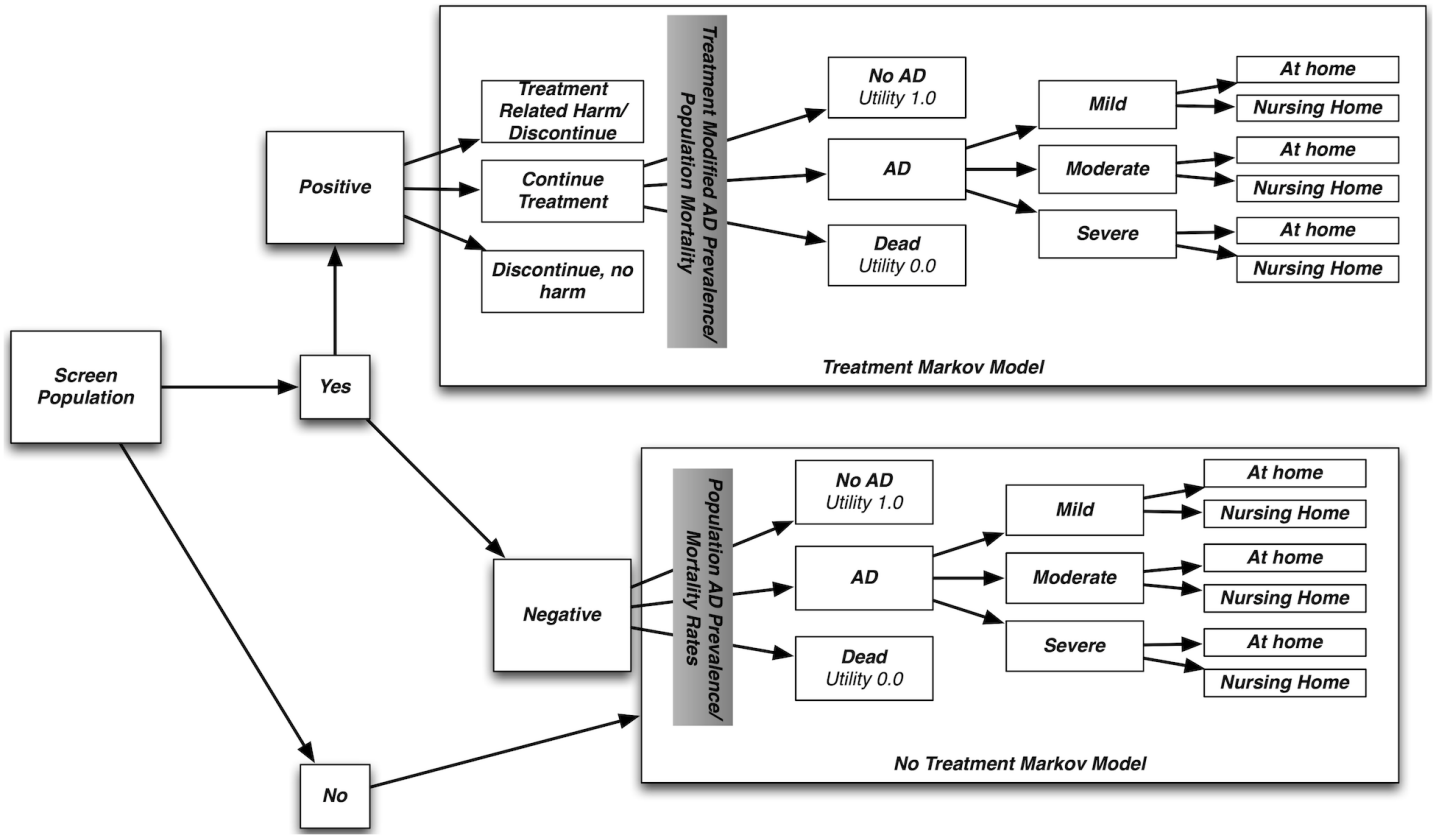
State change (Markov) model. How patients were selected for either the treatment or the untreated state change models and how state changes were assigned annually. Severity and living at home were hierarchically structured – once an individual arrived at the lowest level of the hierarchy (severe AD, living in a nursing home) an individual stayed in that state in subsequent years.

Our approach assumes a single pathway to AD that is detectable by the screening strategy. This assumption is consistent with the prevailing amyloid cascade hypothesis that suggests a relatively stereotyped sequence of changes secondary to primary abnormalities in APP metabolism. [3] We designed a Monte Carlo simulation framework that allowed us to estimate the impacts of varying important parameters: age of treatment initiation, screening tool accuracy, overall treatment efficacy, variation in treatment efficacy over time, magnitude and probability of treatment-related harm, and probability of treatment discontinuation. Within this framework, we focused on the trade-offs of the magnitudes of treatment effect and treatment-related harm to estimate the parameters needed to achieve aggregate net population benefit. All simulation parameters are summarized in **Table 1** and the structure of the underlying Markov model is outlined in **Figure 1** **.** All analyses were performed and all simulation code was written in Stata (StataCorp. 2011. *Stata Statistical Software: Release 12*. College Station, TX: StataCorp LP.). Stata do-files will be made available upon request (James Burke; jamesbur@umich.edu).

**Table 1:** Summary of simulation parameters for base case, one-way sensitivity and multi-way monte carlo simulations.

	BaseCaseValue	One waySensitivityanalysisrange	3rd OrderMonteCarloRange	Reference
**Risk of Developing Dementia**				[28]
**Mortality Risk without Dementia**				[18]
				
**Utility without AD**				
Age 55–64	0.872		0.85–0.90	[21]
Age 65–74	0.836		0.81–0.86	[21]
Age 75–84	0.809		0.77–0.85	[21]
Age 85+	0.775		0.72–0.83	[21]
Death Utility	0			
Discount Rate	0.03	0.0–0.25	0.0–0.25	
				
**Utility with AD**				
Mild AD, community	0.37		“+/−0.1”	[30]
Mild AD, nursing home	0.52		“+/−0.1”	[30]
Moderate AD, community	0.18		“+/−0.1”	[30]
Moderate AD, nursing home	0.21		“+/−0.1”	[30]
Severe AD, community	0.02		“+/−0.1”	[30]
Severe AD, nursing home	0		“+/−0.1”	[30]
				
**Initial AD Severity**				
Mild	0.6		“+/−10%”	[22]
Moderate	0.4		“+/−10%”	[22]
				
**Annual Transition Probabilities**				
Mild-to-mild AD	0.614		“+/−10%”	[23], [30]
Mild to moderate AD	0.322		“+/−10%”	[23], [30]
Mild to severe AD	0.042		“+/−10%”	[23], [30]
Mild AD to dead	0.021		“+/−10%”	[23], [30]
Moderate AD to Moderate AD	0.565		“+/−10%”	[23], [30]
Moderate AD to Severe AD	0.339		“+/−10%”	[23], [30]
Moderate AD to dead	0.053		“+/−10%”	[23], [30]
Severe AD to Severe AD	0.847		“+/−10%”	[23], [30]
Severe AD to dead	0.153		“+/−10%”	[23], [30]
				
**Community to Nursing Home Probabilities**				
Mild AD	0.038		“+/−10%”	[23], [30]
Moderate AD	0.11		“+/−10%”	[23], [30]
Severe AD	0.259		“+/−10%”	[23], [30]
				
				
**Intervention Parameters**				
Population Age	55	55–75	55	
RRR temporal slope	0.03	0.0–0.06	Uniform	
RRR ceiling	0.5	0.0–1.0	Uniform	
Treatment Harm Probability	0.001	0.0–0.1	Uniform	
Treatment Harm Magnitude	0.06	0.0–6.0	Uniform	
Treatment Discontinuation Rate	0.05	0.0–0.2	Uniform	
Diagnostic Parameters				
Sensitivity for AD in 20 years	0.65	0.50–1.0	Uniform	
Specificity for AD in 20 years	0.95	0.50–1.0	Uniform	

### 2.1 Study Population

Using the best available data, we developed a series of simulated patient populations.^25,26^ Each population represented one million adults without AD, but at risk for developing AD at ages varying in five-year increments from 55 to 75. For each individual, the age at which the patient died, whether the individual developed AD, and the age of AD onset were assigned to ensure a realistic population distribution of mortality and AD.

For each individual in each simulated population, we developed a life-history – for each year after the baseline, patients were assigned to one of three states: 1. No AD; 2. AD; or 3. Death. States were assigned such that, on average, the population’s incidence of AD and the probability of mortality mirrored the best available data on the United States population.

First, we randomly assigned gender to each individual in our population based on the base age and data from the US census such that the proportion of each sex in our population mirrored the US population. [17] Next, we separately modeled mortality in individuals who had not developed AD and patients who had developed AD. For individuals without AD, we relied on life table data from the Centers for Disease Control and Prevention (CDC). [18] These data estimate, by gender, the probability of dying at a given age. We applied these data to our population by generating a random variable representing each year of possible life from the base age to age 120. For each year, this random variable represented a probability of death between 0 and 1. The individual was estimated to die in that year if that random variable was less than the proportion of the population at that age that dies in a given year, by sex. Conversely, for individuals after developing AD, mortality was estimated using the state transition model outlined below. (Section 2.2).

We used a similar approach to assign whether an individual develops AD in a given year. The population-based incidence of AD was based on the equation of Brookmeyer et al. [19] This equation estimates AD in a population as low as age 60. To estimate incidence in populations with lower ages, we used linear interpolation such that for every 5 years of decline in population that the incidence would decline by half. Each patient was assigned a random variable between 0 and 1 representing their overall risk of converting to AD. Based on this risk variable, the “highest risk” proportion of the population (regardless of whether they were still alive) – those with the highest risk variables that had not yet converted to AD – was assigned to convert to AD. The number of “highest risk” individuals that converted was given by the Brookmeyer equation.

### 2.2 State Transition (Markov) Model

A simple state transition model was developed for all modeled individuals to estimate net utility, measured in quality-adjusted life years (QALYs) for all individuals in the sample population. This approach uses discrete stages of disease severity and progression to represent both variation in the overall population and changes in individuals over time. While a continuous distribution of stages would have been preferable, we opted for this discrete modeling approach because utilities were available for these discrete stages and thus we were able to make overall measurements in QALYs to enable comparison to other disease states [20]. For individuals without AD, a simple state transition model was used that assigned utility based on age group. [21]. For individuals with AD, a Markov-based state transition model was used to estimate their utility, measured in quality adjusted life years (QALYs). The structure of this model was based on the model of McMahon et al [22] and is summarized in **Figure 1** **.** For individuals that developed AD, they were first randomly assigned an initial disease severity – mild, moderate or severe, in proportion to the population data. For each subsequent year that the patient survived with AD, they were then assigned a new severity (e.g. convert from mild to moderate) based on population-based data on state transitions. After assigning severity to each patient, whether the patient lived in a nursing home or at home was assigned based on severity and following published state transition probabilities. [23] For each combination of severities and locations, whether the patient died within a given year was estimated using a similar approach. Utility was assigned to AD survivors for each possible combination of severity and living location. For surviving individuals without AD, utilities were assigned for every year they survived using age group-based assignments. [21] No additional treatment was modeled in patients with AD, as the effects of existing therapies on quality of life (e.g. donepezil) were judged to be small by comparison to the efficacious presymptomatic therapies modeled in this study. All future utilities were discounted at 3% per year from the base age. For an individual alive without AD at age 90 after being screened at age 55, for example, the utility for the age 90 year was discounted by (1–0.03)∧(90–55) = 0.34.

### 2.3 Modeling Treatment Effects

To inform presymptomatic screen and treat strategies, we modeled a wide variety of treatment effects. We pursued two primary questions – the impact of treatment efficacy and the importance of time of treatment initiation relative to onset of disease. We modeled two components of treatment efficacy – relative risk reduction (RRR): 1. the temporal slope of increasing treatment efficacy (RRR temporal slope) and 2. the treatment effect ceiling (RRR ceiling). RRR ceiling represents the maximum treatment effect size, regardless of when treatment was initiated relative to disease onset. This approach is based on two concepts; even highly efficacious treatments will fail in some patients and the magnitude of treatment benefit increases with earlier treatment. The second concept derives from the biomarker literature indicating that AD pathology begins to develop many years before patients become symptomatic. [24] As a consequence, treatment is likely to be more efficacious the earlier it is initiated.

RRR temporal slope represents changes in treatment efficacy when a treatment is initiated earlier or later relative to disease onset. RRR was then defined as RRR slope multiplied by years from treatment initiation to projected AD onset in the absence of treatment, with the magnitude of RRR capped at the RRR ceiling. Assuming an RRR slope of 0.03 and RRR ceiling of 0.5, for example, a patient destined to develop AD 10 years after treatment initiation would have a RRR of 0.3 (10 years x RRR slope of 0.03 = 0.3, without reaching RRR ceiling) and a patient destined to develop AD 30 years after treatment would have a RRR of 0.5 (30 year * RRR slope of 0.03 = 0.9, which is over the RRR ceiling and thus the ceiling is applied).

We separately modeled three other treatment relevant parameters; probability of treatment-related harm, magnitude of treatment-related harm, and probability of medication discontinuation. For each treated individual, we assumed a fixed annual probability of harm of a fixed QALY decrement. (e.g. a 0.1% risk of a 0.06 QALY loss due to treatment). For individuals that were harmed, we assumed that they discontinued treatment permanently at the time of harm and did not receive any future treatment benefit. We separately modeled a fixed annual probability of medication discontinuation independent of the probability of harm to account for the fact that some patients are likely to discontinue medication for non-harm related reasons [25].

### 2.4 Modeling Intervention Diagnostic Parameters

Presymptomatic treatment strategies implies either treating all patients (for example at a set age) or treating patients with a probability of AD above a threshold defined by clinical data and diagnostic testing data. (e.g. using a clinical risk prediction algorithm based relying on screening tests). Given that tools to select high-risk patients (e.g. ligand-based PET scanning, CSF analysis, genome-based prediction strategies) have promise, our simulations assumed a screening strategy, although we also modeled a treat-all strategy as a comparison. Our simulation assumed that a screen would predict risk of conversion to AD in 20 years. Using specified sensitivities and specificities and the subject’s pre-test 20-year probability of developing AD (the population-average for their age), we determine the expected proportion of true and false test results. To estimate AD outcomes in the absence of screening and treatment, each individual was assigned a random variable specifying the likelihood of accurate classification and was used to assign individual patients to diagnostic categories (i.e. true positive, false positive, true negative, false negative). To estimate AD outcomes if patients were screened and treated, it was assumed that both true and false positive patients underwent treatment and had reductions in their probability of converting to AD based on the treatment’s RRR temporal slope and ceiling.

### 2.5 Monte Carlo Simulation

We used Monte Carlo simulation [26] to estimate outcomes under a variety of treatment scenarios while accounting for uncertainty in AD conversion rates and the likelihood of mortality by repeatedly selecting trial populations from the overall study population. Treatment scenarios were defined by the complete set of input parameters. Our base-case scenario represented a highly effective therapy: baseline population age = 55; RRR temporal slope = 0.03; RRR ceiling = 0.5; probability of harm per year = 0.001; magnitude of harm in QALYs = 0.06; probability of discontinuation independently of harm per year = 0.05; screening sensitivity = 0.65; screening specificity = 0.95. In this scenario, steady-state maximum efficacy would be achieved for individuals that initiate treatment 17 or more years earlier than they would otherwise develop AD. The base-case probabilities of harm [1], [15] and discontinuation [27] parameters roughly parallel the characteristics of aspirin in primary prevention of cardiovascular disease, and were assigned randomly based on these rates.

To estimate population net utility, we applied these screening and treatment parameters to a set of individual patients and compared outcomes (in QALYs) with the same set of patients without screening or treatment. In addition, we separately estimated the change in outcomes associated with treating all patients regardless of their results on screening tests. To account for uncertainty of the baseline risk of conversion to AD and the risk of mortality, we performed a series of 1,000 trials where 1,000 random individuals were selected from our overall study population and the utility per year was estimated. Net utility was estimated as the mean net utility over all trials.

This modeling approach does not directly account for some of the factors that may influence both the transition to AD and progression with AD (e.g. vascular risk factors, education, physical activity, comorbid illnesses). However, as the primary model parameters are drawn from population-based studies, by modeling variation around these parameters, our approach should account for the effects of unmodeled factors. So, while our approach does not enable inferences about sub-populations with unmodeled factors, the estimates for the entire population should not be affected by omitting these factors.

### 2.6 Sensitivity Analyses

As no treatment has yet been shown to effectively treat presymptomatic AD, we examined the net benefit under a wide variety of hypothetical treatment scenarios by varying base-case parameters. Initial one-way sensitivity analyses varied primary model parameters (population age at screening, treatment efficacy parameters, treatment harm parameters, and probability of discontinuation) over a wide spectrum of possible values. To further examine benefit-harm trade-offs, we performed a two-way sensitivity analysis that varied the magnitude and probability of harm over a range of values holding the other parameters at their base-case values.

To explore how treatment parameters interact we also performed a multi-way probabilistic sensitivity analysis (3^rd^ order Monte Carlo simulation) where all parameters (treatment effects, diagnostic parameters and state transition parameters) were varied across a range of plausible values. While the base-case assumptions of our overall modeling approach were selected to reflect a specific scenario, the amyloid-cascade hypothesis of one pathophysiologic pathway to neurodegeneration in AD identified via a screening test, it is possible to interpret model findings independently of that pathway. Any presymptomatic test and treatment approach will necessitate decisions, even if implicit, about who should be treated that will be reflected in the wide range of sensitivies and specificities explored in sensitivity analyses. Similarly, even if the amyloid-cascade hypothesis is invalidated, given the wide range of general treatment parameters explored it will likely be the case that our models will reflect the range of potentially efficacious therapies. This analysis was performed by repeating our baseline simulation while varying all parameters of interest. With each simulation iteration, all simulation parameters were randomly drawn from the probability distributions outlined in Table 1. Descriptive statistics were used to summarize parameters of interest comparing simulation iterations that resulted in net societal benefit to those that did not result in net societal benefit.

## Results

### 3.1 Population and Treatment Assumption Validation

**Figure 2:**
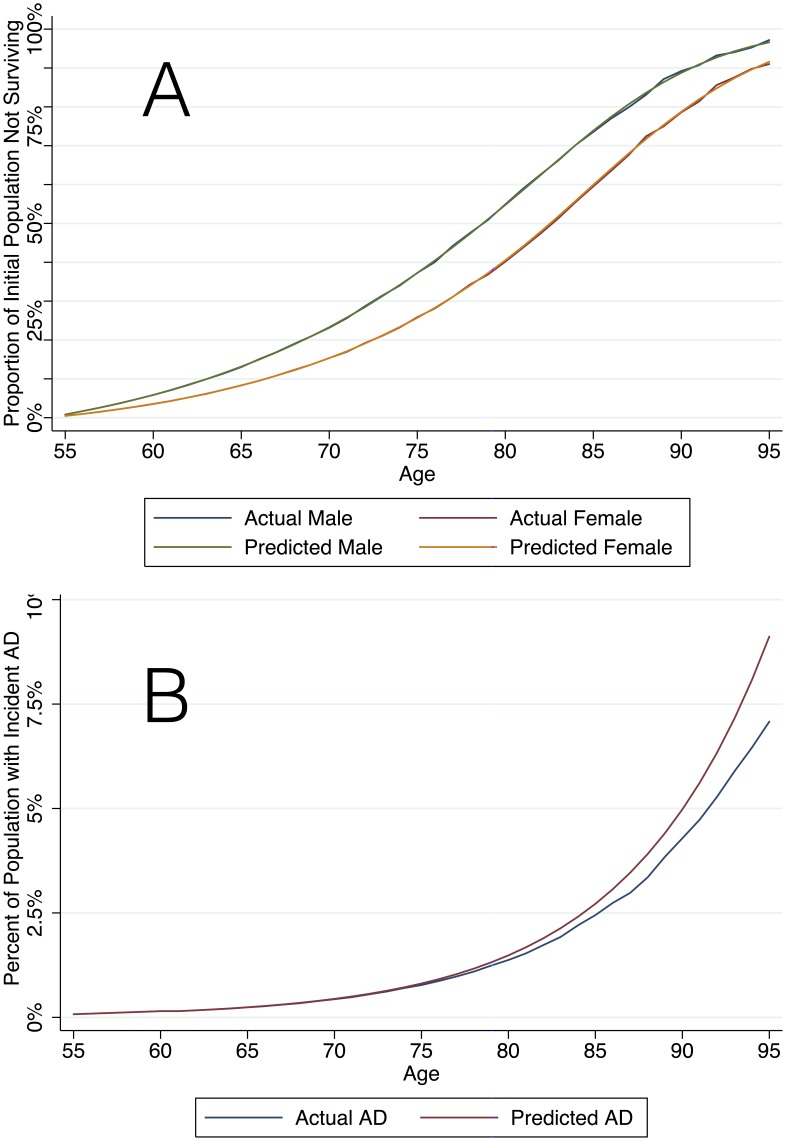
Study Population Validation. Panel A demonstrates the proportion of the initial population surviving at each age group in the simulated population (“Actual”) compared to CDC life table data (“Predicted”) and Panel B demonstrates the proportion of the simulated population with incident AD (“Actual”) compared to the Brookmeyer estimates (“Predicted”).

**Figure 3:**
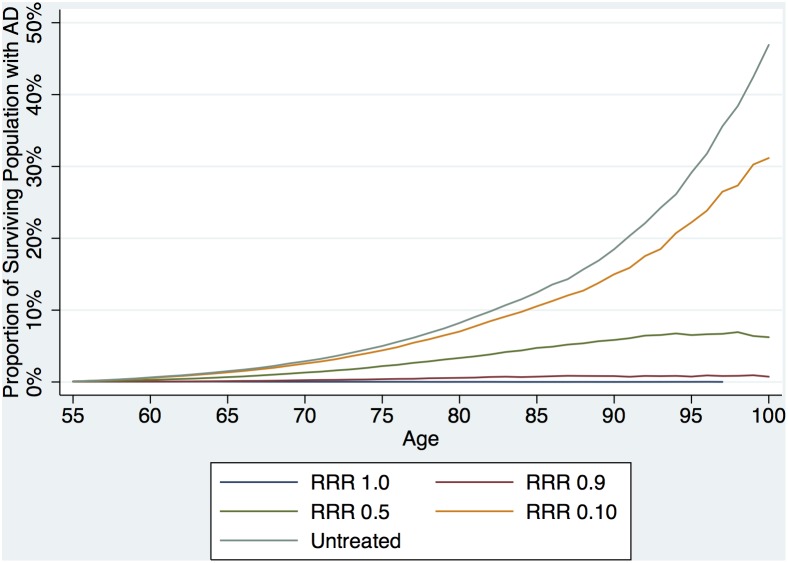
Treatment Effect Validation. Each line represents the proportion of surviving population with AD (estimated prevalence) as the treatment effect size varies assuming all individuals are treated, individuals never discontinue treatment and there is no heterogeneity of treatment effect.

**Figure 2A** demonstrates age-related and gender-specific mortality in our base-case scenario without screening or treatment. In addition, **Figure 2B** demonstrates the estimated incidence of AD by age compared to the Brookmeyer et al equation. [19] **Figure 3** displays estimated prevalence of AD by age for a series of simple intervention scenarios (no heterogeneity of treatment effect, no treatment related harm, no treatment discontinuation) where only the relative risk ceiling, and thus the effective relative risk reduction, was specified. A RRR of 0.5 has a marked effect on AD prevalence.

### 3.2 Base-Case Analyses

In this base-case scenario, the net utility of screening/treatment compared to no screening/treatment was consistently positive (**Table 2**). [1] The net benefit *per 1000 patients screened* was substantial higher for older populations (52 QALYs saved in 75 year-olds vs. 20 QALYS saved in 55 year-olds). The greater benefit per patient screened in older populations was mainly due to the higher probability of being treated – per 1000 screened, 370 75 year olds were treated vs. 89 55 year-olds. However, the benefit *per patient treated* was even higher for younger patients than older patients (221 QALYs saved per 1000 55 year-olds treated vs. 142 QALYs saved per 1000 75 year olds treated.

**Table 2:** Outcomes of base-case scenario for different age groups.

		Screen and Treat if Screen Positive					Treat All				
		Age					Age				
		55	60	65	70	75	55	60	65	70	75
**Overall Treatment Effect**	**Net societal utility**(QALY/1000 personsscreened)	20	28	38	47	52	59	72	84	92	93
	**Net societal utility**(QALY/1000 personstreated)	221	240	227	188	142	59	72	84	92	93
**Diagnostic** **Outcomes** **and Benefit** **Distribution**	**Number** **patients** **treated** (Per1000 patientsscreened)	89	118	169	251	370	1000	1000	1000	1000	1000
	**Number** **false** **positives** (Per1000 patientsscreened)	47	44	40	33	24	936	887	801	665	469
	**Net utility in** **all false** **positives** **(**QALY/1000patientsscreened)	1.6	1.4	1.1	0.8	0.3	32	30	25	16	8

This base-case scenario represented a best-case scenario for presymptomatic screening/treatment – such that there was even modest net benefit even in “false positive” patients. This apparent paradox was due to the definition of sensitivity and specificity used in this study, which was the probability that an individual patient would develop AD 20 years subsequently. Two patients screened positive at age 55 and subsequently converting to AD at ages 70 and 80, respectively, would be classified as a “true positive” and a “false positive.” So, “false positives” identified at age 55 on the whole had a small treatment benefit over the course of their life (0.03 QALYs gained per individual), they had a small net harm at age 75, (0.009 QALYs lost per individual). Given the large benefits and modest harms of therapy in the base-case scenario, the aggregate benefits in false positives eventually converting to AD (e.g. the 80 year old) was slightly greater than the aggregate harms associated with treating false positives not developing AD.

Analogous effects were found in comparing the base-case scenario with a treat all strategy. At age 55. for example, a total of 52 QALYs per 1000 patients treated were generated with a treat-all strategy compared to a screen and treat approach. While this was greater than the 20 total QALYs generated by screening and treating at age 55, the benefit per patient treated was considerably greater for the screen and treat approach compared to the treat all strategy (221 QALYs vs. 52 QALYs).

### 3.3 One-way Sensitivity analyses

**Figure 4:**
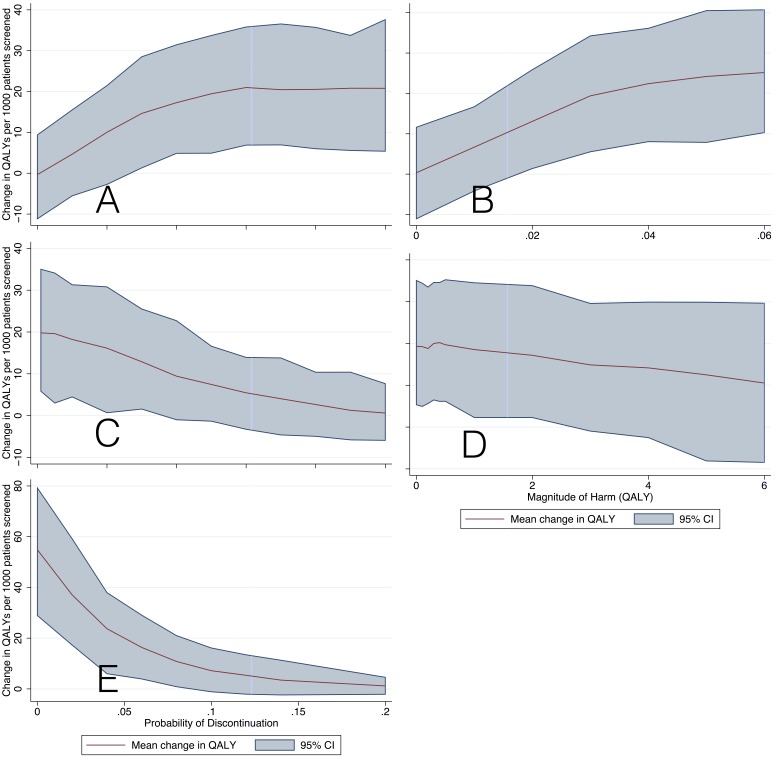
One-way sensitivity analyses. The y-axis for each panel displays the change in the number of expected QALYs with the implementation of a treatment intervention vs. no intervention in a 55 year-old population as treatment efficacy, harm and probability of discontinuation are varied across the x-axis. A) RRR ceiling B) RRR slope C) Probability of Harm D) Magnitude of Harm E) Probability of Discontinuation.

These analyses demonstrate how net benefit changed as each primary parameter of interest was varied (**Figure 4** **).** As expected, both treatment efficacy parameters (treatment RRR ceiling and temporal slope) are quite influential, with a nearly linear increase in net benefit as efficacy increases and plateauing at very high levels of efficacy. Given the low treatment-related harm assumed in our base-case, even modestly effective therapies (e.g. RR 0.95) produce net benefits, however, there is a steep decline in net benefit as treatment-related harm increases. Still, as long as an AD preventive treatment is highly effective, a net benefit persists even at a relatively high probability of harm – as high as 10%/year. Medication discontinuation had a marked effect on net utility, subjects discontinuing treatment incurred the short-term risks of harm but treatment benefits require long-term treatment. Net benefit falls by almost half when the discontinuation rate reaches 3% per year and half again at 6% per year.

### 3.4 Two-way sensitivity analysis

**Figure 5:**
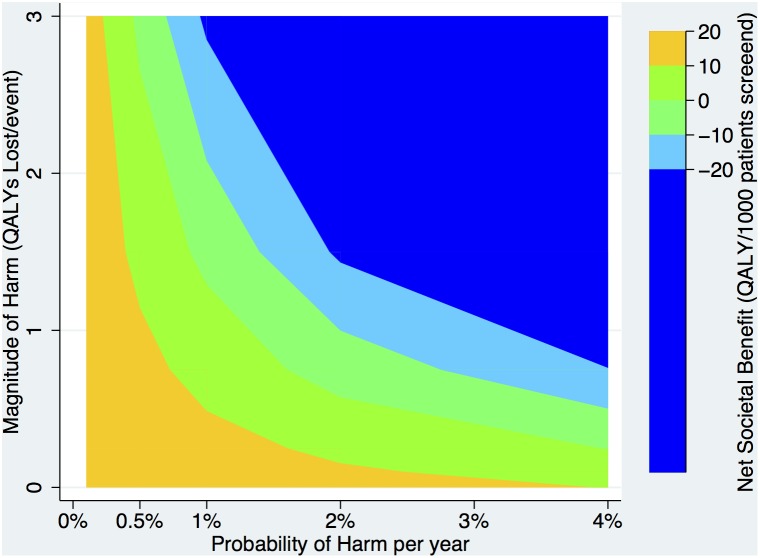
Two-way sensitivity analysis: Magnitude vs. Probability of Harm. The net societal benefit in QALYs/1,000 patients screened is indicated by the colors/shades in the two-dimensional panel as the probability of harm per year is increased across the x-axis and the magnitude of harm (in QALYs/event) along the y-axis.

Given that the base-case scenario represents a highly favorable scenario (good efficacy and low treatment-related harm), we explored the interaction of these two factors in a two-way sensitivity analysis. We found that even when harm was relatively probable (4%/year), aggregate net population benefit persisted when treatment-related harm was moderate (0.3 QALYs lost per adverse event). Similarly, net benefit persisted for relatively uncommon (1%/year) but very severe adverse events (2 QALYs lost per adverse event) in the context of highly effective therapies. (**Figure 5** **).**

### 3.5 Multi-way sensitivity analysis

**Figure 6:**
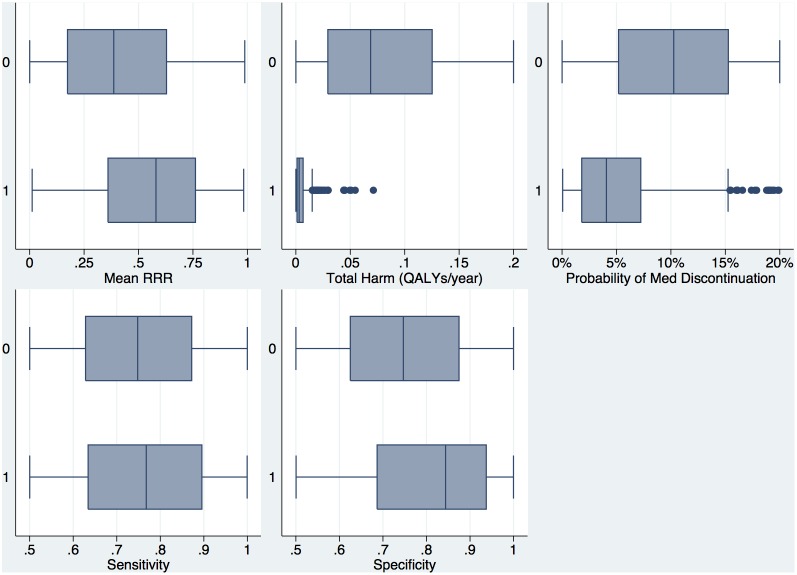
Multi-way sensitivity analysis: Distribution of Intervention Parameters in Beneficial vs. Non-beneficial trials. Box plots of the distribution of intervention parameters across the range of parameters studied. X-axis for each individual plot represents the range of parameter values. Total harm represents the combination of the probability and magnitude of harm in average total QALYs/year lost to harm in treated patients. This parameter was truncated at a value of 0.2 (one QALY lost for every five patient-years) so that the distribution in beneficial trials could be more clearly seen.

Out of 10,000 simulations, 318 resulted in a net societal benefit. Parameter distributions in the trial simulations that resulted in net societal benefit are compared to those that did not in **Figure 6** **.** Across the speculative range of parameters included in this analysis, the total harm in QALYs per year (probability of harm * magnitude of harm) was the most important parameters as 75% of beneficial trials had a total harm of less than 0.007 QALYs per year and 95% had total harm less than 0.02 QALYs/year. Treatment effect was also an important parameter as the mean relative risk reduction in beneficial trials was 0.55 vs. 0.41 in non-beneficial trials. Discontinuation rates were lower in beneficial trials (mean rate 5.4% vs. 10.1%) and specificity was higher (mean spec 81% vs. 75%). Sensitivity was only modestly higher in beneficial vs. non-beneficial trials (76% vs. 75%). For mean relative risk reduction, discontinuation rates, sensitivity and specificity, simulations with net societal benefit existed across nearly the entire range of parameters explored.

**Figure 7:**
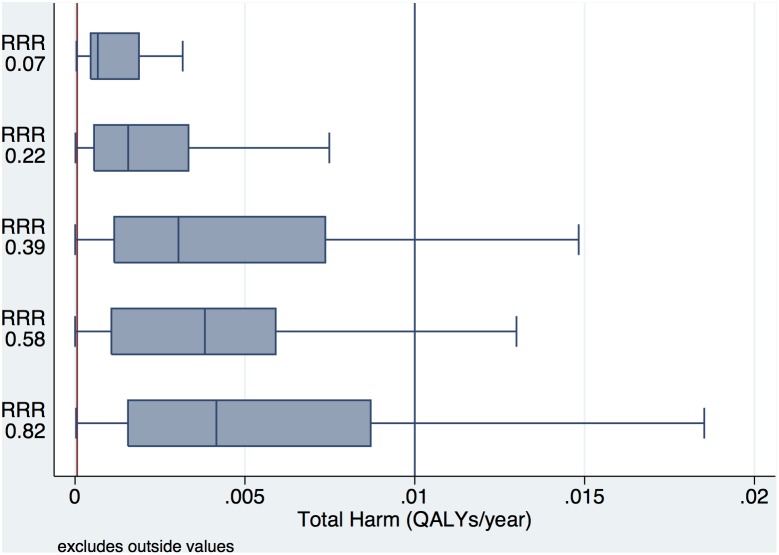
Multi-way sensitivity analysis: Relationship of Treatment Effect and Average Harm. Each box plot represents the distribution of total harm (probability of harm times the magnitude of harm  =  totally QALYs/year lost to harm in treated patients) across quintiles of treatment benefit, represented with the average relative risk reduction in that quintile. The least beneficial simulations are displayed at the top (RRR = 0.07) and the most beneficial trials at the bottom (RRR = 0.82). The red line represents the approximate net harm of aspirin per year (probability of harm = 0.001, magnitude of harm = 0.06 QALYs) and the blue line represents a theoretical medication with a more harm profile (probability of harm = 0.001, magnitude of harm = 10 QALYs or probability of harm = 0.01, magnitude of harm = 1 QALY).

To inform potential drug development, the relationship between quintiles of total treatment benefit and total harm (probability of harm multiplied by the magnitude of harm) is outlined in **Figure 7**. As treatment benefit increases, more harmful treatments are potentially consistent with net societal benefit. For medications with a risk as low as aspirin (illustrated by the red line in the figure) a significant number of net beneficial simulations existed even in the least beneficial quintile of total treatment benefit (range of mean relative risk reduction 0.01– mean relative risk reduction 0.14).

## Discussion

Implementing presymptomatic AD screening and treatment approaches entails substantial challenges. Current AD risk prediction is limited and it is uncertain if potentially harmful long-term treatment of presymptomatic individuals will yield net benefits. Our modeling suggests that efforts to develop new screening tools and to conduct AD prevention trials may be worth the effort, if relatively safe and effective tools can be identified. In particular, we found that a moderately effective treatment should produce substantial net benefits even in the face of relatively frequent or severe adverse events. Even if an effective therapy must be started many years in advance, the benefits per patient treated would likely be greater than the benefits of aspirin therapy for primary prevention of cardiovascular events. In our base case scenario, the benefit accompanying screening and treating 55 year-olds (221 QALYs) was almost 10 times greater than the estimated net population benefit of aspirin for primary cardiovascular prevention in moderate cardiac risk men (28 QALYs/1000 treated) and even greater than the benefit in very high risk men (175 QALYs/1000 treated) [1].

These findings were partly a reflection of the optimistic parameters embodied in the base-case scenario – a highly effective AD treatment as safe as aspirin. However, we found that only modest efficacy of the AD prevention treatment is needed as long as the treatment is relatively safe (comparable to aspirin) and treatment adherence was high. For most treatments, aggregate net population benefit (hereafter, net benefit) that is merely above zero is a very low standard as treatment costs are important for subsequently establishing relative cost-effectiveness. For presymptomatic AD screening and treatment, a relatively low bar is justifiable given the enormous societal costs of long-term care associated with AD, the projected growth in these costs and the unique societal value of developing effective therapies when existing therapies have limited benefit. These optimistic base-case parameters also resulted in the an apparently paradoxical finding of a very small net benefit when treating patients who were “false positives” and a positive net societal benefit even when treating all patients in the absence of a screening test. A potential implication of this finding is that for very safe and inexpensive treatments, accurately identifying patients may not be essential for delivery of net benefits.

While our primary base-case parameters were favorable for the presymptomatic screening/treatment paradigm, other elements of our base-case scenario may minimize the potential utility of an effective therapy. Our modeling scenarios incorporated one-time screening at progressively rising ages. Given that net benefits were greater, per patient treated in younger patients, repeated screening strategies for low-risk treatments may result in even greater net benefits. In addition, it is probable that therapies reducing AD incidence would slow progression of AD, an element not considered in our current model and which may yield additional net benefits [28].

Our finding of higher net social benefit when screening and treatment were initiated at older ages was somewhat surprising given that our modeling approach explicitly increased treatment efficacy when targeting younger patients. With the exponential increases in AD incidence over time, however, older populations have more patients at high risk for converting to AD and our model found that the number of treated patients increases substantially with screening and treating older cohorts. Even though the incremental gain per-treated patient was smaller in older populations, this was more than offset by increases in the number of treated patients. This is a typical result in utilitarian valuation systems where benefits are aggregated across large participant numbers. [29] This phenomenon raises the difficult question of balancing greater individual benefits to smaller numbers of subjects with small benefits accruing to larger numbers of subjects. It is likely that this trade-off will be most stark when considering the cost effectiveness of pre-symptomatic screening and treatment approaches. Earlier treatment implies screening larger populations, longer duration of treatment and a longer period of exposure to risk – all of which will result in increased costs compared to later screening. While it is premature to undertake formal cost-effectiveness analyses without demonstrably efficacious therapies with reasonably quantifiable risks, broad–based modeling and cost-benefit analyses may inform therapy development by helping target therapies and selecting between screening approaches. Ultimately, if and when such efficacious therapies emerge, costs of therapy will be only one of many important societal issues that will demand attention with anxiety from false positives, harms of screening tests and patient and provider incentives towards overtreatment amongst them.

Our analyses suggest an important effect of the probability of treatment discontinuation on net benefits. The aggregate net population benefit of presymptomatic AD screening/treatment would be substantially attenuated, with an approximately three-fold decline in predicted net benefits, by discontinuation rates of 5%/year and an approximately six-fold decline in predicted net benefits for discontinuation rates of 10%/year. These discontinuation rates are consistent with estimates of discontinuation rates for prophylactic anti-platelet and anti-hypertensive therapies. [27] Medication compliance is sensitive to numerous factors, including regimen complexity, minor harms such as cosmetic side-effects, and costs. One possible implication of our analyses is that a very successful presymptomatic AD therapy is one that would not require chronic administration and possess high efficacy. A therapy with these characteristics would be successful even in the face of significant, albeit rare, treatment associated harms. Active immunization against Aß peptide species, which has largely been abandoned, may have these characteristics. Whether or not individual patients would opt for such treatments is an open question.

In summary, our analyses suggest that effective presymptomatic AD screen/treat strategies should produce large net social benefits, even if benefits take many years to accrue and the treatment has substantial adverse effects. Our results emphasize the importance of treatment efficacy and suggest that maximizing treatment efficacy should be a major goal of clinical trials. Our analyses also identify regimen compliance as a critical issue for successful implementation of presymptomatic AD therapies, a problem that should be considered early in the drug development process. Modeling is likely to be useful in guiding development of screening strategies, clinical trial methodology, and cost-benefit analyses for presymptomatic AD treatment strategies.
